# Contemporary Evolution of an At‐Risk Stickleback Population During a Severe Drought

**DOI:** 10.1111/eva.70189

**Published:** 2026-01-06

**Authors:** Sarah Sanderson, Lucas Eckert, Rowan D. H. Barrett, Thomas E. Reimchen, Andrew P. Hendry

**Affiliations:** ^1^ Department of Biology and Redpath Museum McGill University Montreal Quebec Canada; ^2^ Department of Biology University of Victoria Victoria British Columbia Canada

**Keywords:** adaptation, contemporary evolution, environmental disturbance, *Gasterosteus*, genomics, intraspecific variation

## Abstract

Populations can be granted conservation status because they harbour a set of unique traits, evolutionary histories, or ecological roles. Such populations are often isolated and specialised and, as such, can be particularly vulnerable to environmental disturbances. Even if distinct populations survive and adapt to severe disturbances, they could show changes in the very traits that made them distinct in the first place. Here, we leverage a natural ‘experiment’ involving an unarmoured population of threespine stickleback ( *Gasterosteus aculeatus* ) in Rouge Lake (Haida Gwaii, BC)—a population listed as Special Concern under the Canadian Species at Risk Act. In 2015, Rouge Lake nearly dried up during a severe drought event; yet the stickleback population appeared to have fully recovered its abundance in subsequent years. Using phenotypic measurements, we assessed the extent to which evolution in this population was impacted by the drought. We document important shifts in several phenotypic traits, with the largest occurring in precisely the trait that made the population distinct and prompted its original conservation designation. Specifically, fish with no lateral plates (i.e., ‘unarmoured’) made up 51% of the population before the drought but only 13% after the drought. This shift held (13%–16% unarmoured) over the 4 years of our post‐drought monitoring. Field observations support a strong demographic bottleneck, which we suggest might have been coupled with a shift in the selective regime. These findings underscore how populations of conservation concern are not only at risk of extinction; they are also at risk of losing the characteristics that make them unique. These dynamics highlight the need for policies to consider a population's evolutionary potential and develop more flexible approaches than simply considering single‐timepoint assessments of diversity.

## 1 Introduction

Human influences have accelerated biodiversity losses far beyond background levels (Ceballos et al. 2015; Ceballos and Ehrlich 2002; Dirzo et al. 2014). Much of the focus on these losses has emphasised species extinctions; yet diversity within species is also of increasing concern (Ceballos and Ehrlich 2002; Mimura et al. 2017). Specifically, humans are causing population extinctions (Ceballos et al. 2015; Ceballos and Ehrlich 2002; Dirzo et al. 2014), changes in population mean phenotypes (Oke et al. 2020; Sanderson et al. 2021), as well as changes in within‐population phenotypic variation (Sanderson et al. 2023) and genetic diversity (Leigh et al. 2019; Shaw et al. 2025). Such changes in intraspecific diversity are concerning because they can reduce evolutionary potential (Mimura et al. 2017; Pauls et al. 2013), compromise portfolio effects (Schindler et al. 2010), and alter community structure and ecosystem function (Hendry 2017). Indeed, intraspecific variation (e.g., different genotypes) can have community and ecosystem effects as large as inter‐specific variation (e.g., different species) when tested in experimental arenas (Des Roches et al. 2018).

Such concerns about decreasing intraspecific diversity are increasingly finding their way into the policy decisions of management agencies and governments (Hoban et al. 2021). In particular, conservation efforts now often focus below the species level by designating particular populations as units of conservation—typically with the goal of conserving unique evolutionary histories, ecological roles, genotypes, or traits (Coates et al. 2018). As one example, the US Endangered Species Act (ESA) allows for the designation of Distinct Population Segments (DPS) that are ‘substantially reproductively isolated from other conspecific populations and that represents an important component of the evolutionary legacy of the species’ (Waples 1991). As another example, the Committee on the Status of Endangered Wildlife in Canada (COSEWIC) recognises populations (or groups of populations) within species as Designatable Units (DUs) that are both discrete—meaning ‘currently very little transmission of heritable information from other unit’—and evolutionarily significant—meaning that ‘the unit harbours heritable adaptive traits or an evolutionary history not found elsewhere in Canada’ (COSEWIC 2023a, 2023b, 2023c; Mee et al. 2015). Many of these populations are then formally protected under the Canadian Species at Risk Act (SARA) (Hutchings and Festa‐Bianchet 2009). Related approaches to the conservation of populations are implemented in many other countries and regions (Coates et al. 2018).

The legalised protection of specific populations within a species generally emphasises uniqueness, such as a high frequency of phenotypic traits that are not common elsewhere in the species range. For example, the Peary caribou ( *Rangifer tarandus pearyi* , Allen 1902) is particularly adapted to the desert habitat of the Canadian High Arctic and it is distinguished from other caribou populations in its characteristically smaller stature, densely‐haired whiter pelage, shorter face, larger hooves, and more narrowly spread antlers (Manning 1960). Dramatic population declines (72% over 21 years) thus warranted listing the Peary caribou as Endangered under SARA (COSEWIC 2004). The phenotype‐based designation approach has considerable value because it emphasizes likely adaptive traits; however, it leaves open an important question: What should happen when a listed population remains present at a site, and yet changes phenotypically such that it is no longer unique? Although this scenario might seem unlikely at first glance, some instances have been described.

As one example, the Enos Lake threespine stickleback ( *Gasterosteus aculeatus* , Linnaeus 1758) ‘species pair’ was designated as Endangered by COSEWIC and listed as such under SARA (COSEWIC 2002). The original listing was based on the occurrence of sympatric benthic and limnetic populations that had distinct phenotypes and showed limited interbreeding (McPhail 1984, 1989). Over the last few decades, however, hybridization between the two species has resulted in a unimodal phenotypic distribution and, thus, collapse of the species pair (Behm et al. 2010; Taylor et al. 2006)—perhaps due to interactions with an invasive crayfish (Velema et al. 2012). As the original basis for designation under SARA was no longer met, COSEWIC recommended changing the status of the species pair to Extinct (COSEWIC 2023a, 2023b, 2023c). Their listing under SARA is currently under review as of December 2025. Another relevant context comes from conservation translocations or artificial propagation efforts. For instance, are Florida Panthers ( *Puma concolor coryi* , Bangs 1899) still distinctive enough to warrant listing under the ESA following the introduction of Texas Panthers (*P. c. stanleyana*, Goldman 1938) (Finn et al. 2013; Johnson et al. 2010)? And what should be done if Devil's Hole Pupfish ( *Cyprinodon diabolis* , Wales 1930) placed into refuge habitats evolve noteworthy differences from their ancestral listed population (Wilcox and Martin 2006)?

Evolutionarily distinct populations are typically genetically isolated and specialized to their environment through strong past selection (Coates et al. 2018; Mee et al. 2015; Waples 1991). As such, these populations might be particularly vulnerable to dramatic environmental perturbations such as floods or droughts which have become increasingly common (Easterling et al. 2000; Rahmstorf and Coumou 2011). In such situations, the responses of local populations can range from extinction to sustained low abundances to rapid recovery to previous abundances. In the case of such demographic recovery, the question we consider here is whether the post‐recovery population is substantially phenotypically or genetically different from the pre‐disturbance population. In some cases, the answer is ‘no’, such as when Trinidadian guppy ( *Poecilia reticulata* , Peters 1859) populations are dramatically reduced by floods and yet recover in less than a year without much phenotypic or genetic change (Blondel et al. 2021; Weese et al. 2011). In other cases, however, the answer might be ‘yes’—either because of stochastic effects (e.g., demographic bottlenecks), gene flow (from other populations), or selection. In the present study, we consider a different context under which evolutionarily distinctive traits—traits that formed the basis for legal protection—can change: following the major environmental perturbations of a severe drought event.

### 1.1 Rouge Lake Stickleback

Although threespine stickleback are common across the Northern Hemisphere, some especially distinctive populations have been granted various levels of legal protection, including several species pairs on Vancouver Island (e.g., Enos Lake, as described above), as well as the Giant Stickleback and the Unarmoured Stickleback (both on Haida Gwaii). We here focus on the Unarmoured Stickleback, which are currently listed as Special Concern under SARA (Fisheries and Oceans Canada 2022) but are currently under review after COSEWIC recommended a change in status to Endangered (COSEWIC 2023a, 2023b, 2023c). The morphological uniqueness of these populations was first documented by Reimchen (1984). Their extreme reduction in armour, among the most pronounced cases described across the species' Canadian range (O'Reilly et al. 1993), satisfies the ‘evolutionary significant’ criterion for a DU. Early genetic analyses supported their distinctness (O'Reilly et al. 1993). Subsequent genetic analyses have further demonstrated that the Unarmoured Stickleback are genetically distinct from other stickleback populations (Deagle et al. 1996; Marques et al. 2022) and thus satisfy the ‘discrete’ criterion for a DU. This distinctness and significance are reinforced by the known strong genetic basis for spine loss (Chan 2010; Shapiro et al. 2004) and armour plate loss (Colosimo et al. 2004, 2005). The Unarmoured Stickleback has three independently derived populations in Haida Gwaii: Boulton Lake, Rouge Lake, and Serendipity Lake. Unlike the extensive defensive structures (three dorsal spines, two pelvic spines, an anal spine, and bony lateral plates) found in the vast majority of other stickleback populations, the Unarmoured Stickleback populations lack most defensive structures or express extremely reduced forms of those structures (Reimchen 1984). Because each population is independently derived and exhibits a unique combination of traits that distinguishes it from the other populations, it is possible that this species bundle could constitute as many as three separate DUs (COSEWIC 2024).

**FIGURE 1:**
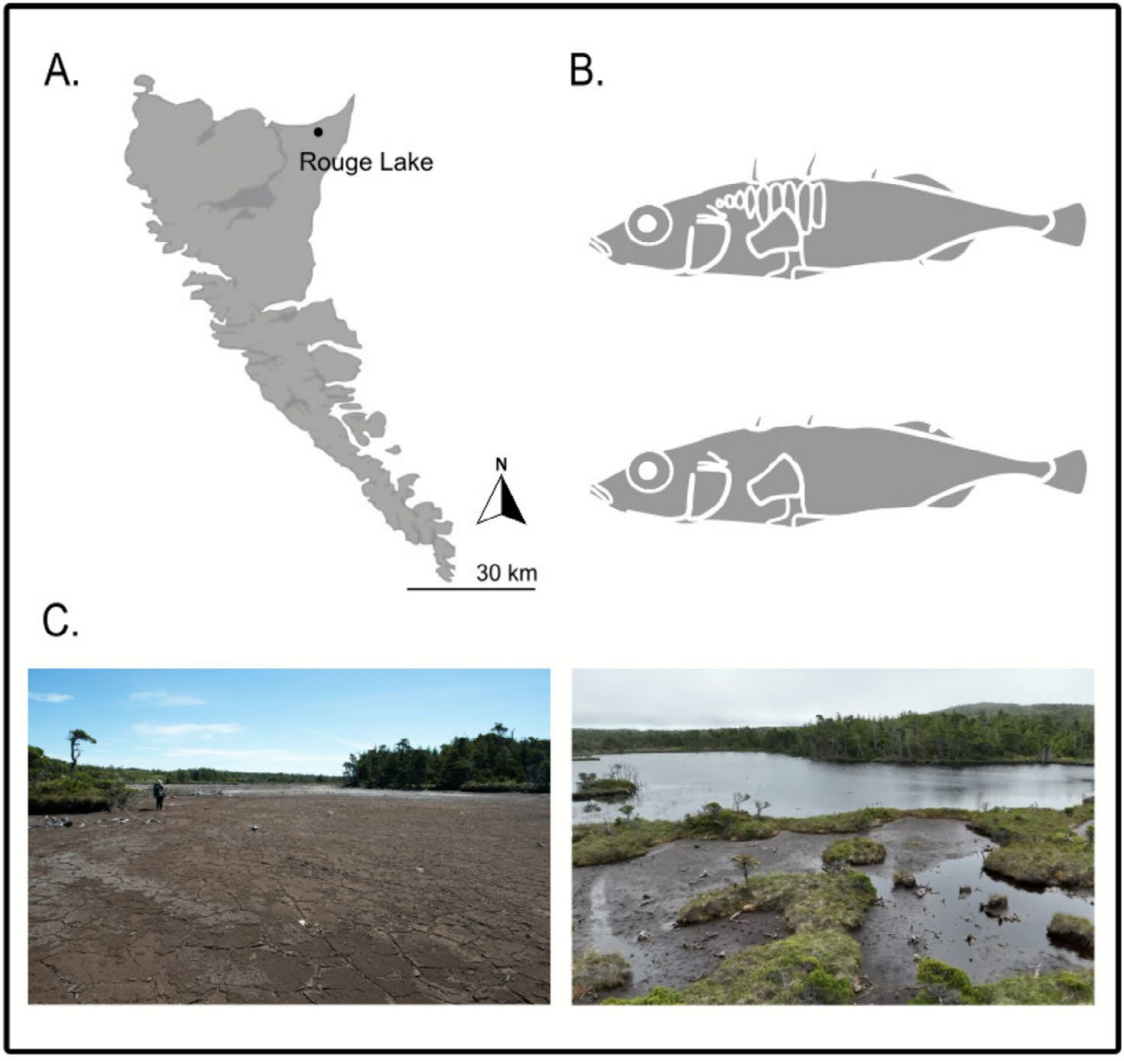
Panel (A) Map of Haida Gwaii showing the location of Rouge Lake. The base map was obtained from iMapBC (https://www2.gov.bc.ca/gov/content/data/geographic‐data‐services/web‐based‐mapping/imapbc) and modified using Inkscape. Panel (B) The top image shows a simplified representation of a common freshwater threespine stickleback, and the bottom image shows a typical Unarmoured Stickleback (from the original Rouge Lake population). Panel (C) The image on the left shows Rouge Lake in 2015 when the drought occurred, and the image on the right shows Rouge Lake in 2022.

Our work considers the effects of a dramatic environmental perturbation on the Unarmoured Stickleback population found in Rouge Lake, Haida Gwaii. Beyond its unusual loss of most armour, this population is unique among all Haida Gwaii stickleback populations in being monomorphic for a rare mitochondrial lineage (O'Reilly et al. 1993) and displays a highly atypical association with a photosynthetic but parasitic dinoflagellate (Buckland‐Nicks and Reimchen 1995). In 2015, this population experienced a severe drought during which the entire lake appeared to dry up (Figure 1), suggesting a potential extirpation. However, a very shallow (< 3 cm) and small (maybe 10 m^2^) puddle in the middle of the lakebed suggested that some fish could have survived. Indeed, after the lake returned to its normal level (the precise date isn't known), a few fish were captured (and released) with approximately the same amount of effort (6–8 traps) in 2016 (*n* = 7) and 2017 (*n* = 6). Then, by 2018 (*n* = 200), the population reached pre‐drought levels (2012, *n* = 150; 2013, *n* = 150) and a sample was collected. This sample contained a number of armoured fish, which inspired the current analysis of whether the population has now evolved away from the characteristics that were the basis of its original designation.

Comparing historical (pre‐drought) and recent (post‐recovery) samples, we ask three questions related to this event:

1. To what extent have phenotypic traits changed following the drought? Here we compare pre‐drought samples to our first post‐recovery sample (2018), considering both the defensive traits that underpinned the original unarmoured listing and additional traits not tied to that listing.
2. Are trait changes from before to after the drought then stable after population recovery? Here we compare the same set of traits between samples collected in 2018 and in 2022.
3. Did the population recover in situ (perhaps from a few fish that survived in isolated pockets of water) or via immigration from elsewhere (perhaps the outlet stream)? Here we used phenotypic measurements and whole‐genome pool‐sequencing to compare samples collected in 2022 from both Rouge Lake and the Rouge Outlet stream.

After answering these questions, we discuss how evolutionary changes in this population—and in populations more generally—could be factored into conservation designation decisions.

## 2 Methods

### 2.1 Study System

Rouge Lake (54.03297° N, 131.87728° W) is located on the northeastern corner of Graham Island (Haida Gwaii) in a sphagnum bog about 2 km from the coast. The lake has a surface area of about 22,000 m^2^, maximum depth of about 2 m and highly acidic water (pH 4.1–4.5). In 1970, Rouge Lake had a sandy shoreline that extended to the edge of the sphagnum banks (Reimchen 1984). In 1975, however, beavers invaded the area and blocked the outlet stream, which resulted in a 0.50–1 m increase in water depth of the lake to its current level. The stickleback population in Rouge Lake (also known as the Charlotte Unarmoured Stickleback) is particularly interesting because of its lack of armour. This rare phenotype is why the population is listed as Special Concern under SARA and as Endangered by COSEWIC.

Although threespine stickleback populations can show substantial differences in armour, the vast majority of populations usually have three dorsal spines, two pelvic spines, an anal spine, and bony lateral plates along both sides of their bodies. The Rouge Lake population, however, is characterised by loss of the third dorsal spine, the anal spine, and the lateral plates (Reimchen 1984). Other unique traits in this population include a reduction in the number of dorsal and anal fin rays, a reduction in size of the cleithrum and pterygiophores, and the development of a postcranial hump (Reimchen 1984). 15% of the fish have two dorsal fins, also an uncommon trait in threespine stickleback (Reimchen 1984). Moreover, Rouge Lake fish have the most divergent body shape out of 118 populations across Haida Gwaii based on geometric morphometric analyses. Their shape is characterised by a thick peduncle, posterior and closely spaced dorsal spines, an anterior pelvis, as well as small dorsal and anal fins (Spoljaric and Reimchen 2007).

Beyond their special phenotypes, several other features make this population noteworthy. First, Rouge Lake stickleback host an endemic species of parasitic dinoflagellate (*Haidadinium ichthyophilum*) only known to occur in that one small lake (Buckland‐Nicks et al. 1990; Buckland‐Nicks and Reimchen 1995; Hehenberger et al. 2018). Second, Rouge Lake stickleback is the only known population in Haida Gwaii to be monomorphic for the Trans‐North‐Pacific mitochondrial DNA lineage (Deagle et al. 1996; O'Reilly et al. 1993). Finally, prior to the beaver invasion, breeding males displayed pronounced red throat pigmentation (the reason for the name of the lake), a feature that has since disappeared (Reimchen 1984). In short, the Rouge Lake population represents a particularly unique component of the diversity of threespine stickleback.

The summer of 2015 saw the most significant drought in recent years across the province of British Columbia (Szeto et al. 2016). By the end of June 2015, the province faced several extreme low‐flow stream advisories, extreme wildfire risks, and water restrictions (AFCC 2016). These exceptionally dry and warm conditions led to some rivers running at their lowest flows since measurements began ~80–100 years ago (CMOS 2016). Southern parts of the province reached Level 4 drought (the maximum level), and the majority of the province was under a Level 3 drought (Province of British Columbia 2015). From June through August, Haida Gwaii was under a Level 3 drought, which included an unusual open fire ban and water restrictions. These conditions led Rouge Lake to almost completely dry up—as noted above.

### 2.2 Field Methods and Trait Measurements

Stickleback were caught using Gee minnow traps in three separate years before the drought (2007, 2012, and 2013) and two separate years after the drought (2018 and 2022). Sample sizes (Figure S1, Table 1) varied among years depending on effort (pre‐drought) or capture rates (post‐drought). In 2022, fish were also caught in the outlet stream to assess the potential for that population to have contributed to the recovery of the lake population (details below). The inlet is very short and small, and prior sampling in the inlet (80s and again in 2022 and 2024) never recovered any fish. We infer that an established population does not exist in the inlet, ruling that option out as a source for recovery of the lake population and leaving the outlet as the only possible source. Stickleback were euthanised immediately using clove oil and then preserved in 95% ethanol. Collections were made in accordance with BC fish collection permits, Park Use permits, and Haida Council permits. Animal handling protocols were approved by the University of Victoria (pre‐drought samples) and McGill University (post‐drought samples). We also measured pH levels in 2002, 2016, 2017, 2018, and 2022.

**TABLE 1:** Numbers of fish analyzed for each year and site. Pre‐drought samples are combined across years.

Location (Year)	Females (*n*)	Males (*n*)	Total (*n*)
Lake (2007‐12‐13)	32	13	45
Lake (2018)	48	40	88
Lake (2022)	39	60	99
Outlet (2022)	14	6	20

#### 2.2.1 Linear Measurements and Meristic Traits

**FIGURE 2:**
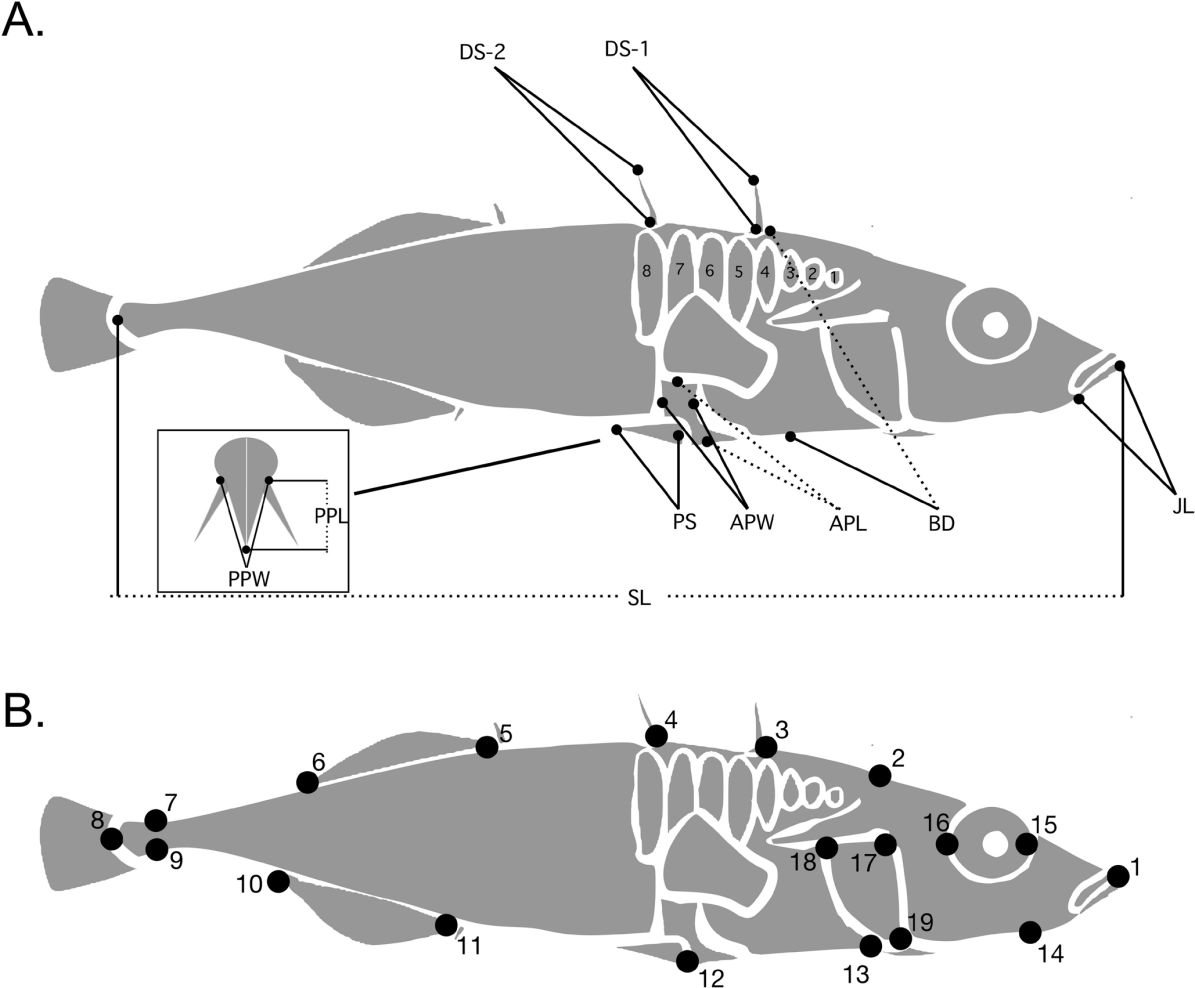
Panel (A). Depiction of the morphological traits measured on a generic threespine stickleback: Standard length (SL), maximum body depth (BD), length of gape (JL), length of the first dorsal spine (D1), length of the second dorsal spine (D2), length of the left pelvic spine (PS), length of the pelvic plate (PPL), maximum width of the pelvic plate (PPW), height of the ascending process (APL), maximum width of the ascending process (APW), and lateral plate count and position (1–8). Panel (B) Landmarks for geometric morphometrics: (1) the tip of the pre‐maxilla, (2) tip of the supraoccipital crest, (3) anterior junction of first dorsal spine and basal plate along the dorsal midline (DML), (4) anterior junction of second dorsal spine and basal plate along the DML, (5) base of the first dorsal fin ray, (6) insertion of the dorsal fin membrane on the DML, (7 and 9) dorsal and ventral anterior extend of the caudal fin membrane, (8) caudal border of the hypural plate at the lateral midline, (10) insertion of the anal fin membrane on the ventral midline (VML), (11) base of the first anal fin ray on VML, (12) insertion of the pelvic spine, (13) anterior border of the ectocoracoid on VML, (14) articular‐quadrate joint, (15 and 16) anterior and posterior dorsal tips of the orbit, (17), anterior dorsal tip of the operculum (18), posterior dorsal tip of the operculum, and (19) ventral tip of the operculum.

We measured 10 linear traits and three meristic or categorical traits (Figure 2) on preserved fish. All traits were measured on the left side of the fish, except plate number and plate position (details below), which were measured on both the left and right sides. The following linear traits were measured to the nearest 0.01 mm using digital calipers: standard length, maximum body depth, length of gape, length of the first dorsal spine, length of the second dorsal spine, length of the left pelvic spine, length of the pelvic plate, maximum width of the pelvic plate, height of the ascending process, and maximum width of the ascending process (Figure 2). Meristic traits scored visually include the number of lateral plates on right and left sides and position of lateral plates of right and left sides (following Reimchen 1983). We also dissected the gonads of each fish to determine sex.

All linear measurements were allometrically standardised for body size according to the equation $M_{\mathrm{std}}=M_{o}{\left(\bar{\mathrm{SL}}/{\mathrm{SL}}_{o}\right)}^{b}$, where *M*_*o*_ is the observed trait length, *SL*_*o*_ is the observed standard length, $\bar{\mathrm{SL}}$ is the grand mean of all standard lengths (51.99 mm) and *b* is the slope of an ANCOVA representing the relationship between *M* and *SL*: log(*M*) ~ log(*SL*) + year (Hendry and Taylor 2004; Reist 1986). Units were then standardised by transforming measurements into standard deviations of the total sample (all fish) and centred around zero (*Z* transformation). Lateral plate number was not adjusted for allometry because plates do not change after fish are > 30 mm in length (Bell 1981, 1987, 2001; Glazer et al. 2014)—as was the case for all of the fish we measured. Indeed, Spearman's rank correlations confirmed that lateral plate number were not correlated with body size for any of the samples.

#### 2.2.2 Geometric Morphometrics

Body shape was quantified using landmark‐based geometric techniques. Digital photographs were taken under consistent lighting with a Nikon D850 camera with a 60 mm lens on a tripod. Landmarks were manually placed at homologous positions on photographs of the left side of the fish (Figure 2) in tpsdig v.2.64 (Rohlf 2006). Landmarks included: (1) tip of the pre‐maxilla, (2) tip of the supraoccipital crest, (3) anterior junction of first dorsal spine and basal plate along the dorsal midline (DML), (4) anterior junction of second dorsal spine and basal plate along the DML, (5) base of the first dorsal fin ray, (6) insertion of the dorsal fin membrane on the DML, (7) dorsal anterior extent of the caudal fin membrane, (8) caudal border of the hypural plate at the lateral midline, (9) ventral extent of the caudal fin membrane, (10) insertion of the anal fin membrane on the ventral midline (VML), (11) base of the first anal fin ray on VML, (12) insertion of the pelvic spine, (13) anterior border of the ectocoracoid on VML, (14) articular‐quadrate joint, (15 and 16) anterior and posterior dorsal tips of the orbit, (17) anterior dorsal tip of the operculum, (18) posterior dorsal tip of the operculum, and (19) ventral tip of the operculum (Figure 2). Missing landmarks (e.g., broken spines) were estimated using *estimate.missing* function from the *geomorph* package. Specimens were ‘unbent’ using the *unbend* function in *tpsUtil* and aligned using landmarks 1, 8 and 18.

#### 2.2.3 Genomics

We conducted whole genome pool‐sequencing on fish from both the Rouge Lake and Rouge Outlet samples from 2022. We also included—as a point of reference—two source lakes and their respective experimental ponds in Haida Gwaii. Mayer Lake stickleback were introduced into Roadside Pond in 1993 (Leaver and Reimchen 2012), and Drizzle Lake stickleback were introduced into Drizzle Pond in 1997 (Planidin and Reimchen 2021; Spoljaric and Reimchen 2007). All sequenced individuals were collected in 2022. Note that DNA in the pre‐drought samples was too degraded to enable sequencing—and so all genetic inferences are from post‐recovery samples.

DNA was extracted from the pectoral fin using a standard phenol‐chloroform protocol. Briefly, tissue samples were placed in digestion buffer containing proteinase K and incubated at 55°C. DNA was then isolated using an isoamyl‐phenol‐chloroform solution, followed by ethanol precipitation. Individual samples were quantified using PicoGreen. These individual samples were then pooled using 100 ng of each individual, with pools based on sampling location and time. Each sample was used to generate a pool of 20 individuals each (i.e., 20 individuals from the lake and 20 individuals from the outlet). Libraries were prepared for these pools and sequenced on a single lane of an Illumina NovaSeq 6000, aiming for coverage of 2X per individual.

We performed quality control on the raw reads (53 M reads for Rouge Lake and 49 M reads for the outlet) using *FastQC* (v.0.12.1) before trimming and aligning them to a reference. We trimmed the reads with *fastp* (v.0.24.0), removing reads with Phred scores less than 20 and lengths less than 50 bp, as well as adapter sequences and polyG tails. The trimmed reads were then aligned using *BWA* v.0.7.18 (Li 2013) to the University of Georgia *G. aculeatus* reference genome v.5 (Nath et al. 2021), and reads with a mapping score less than 20 were filtered out. We sorted and indexed the resulting bam files with *Samtools* (v.1.20) and marked and removed PCR duplicates using *Picard* (v.3.1.0), before converting to an mpileup file. We identified and masked indel regions using *PoPoolation2* (Kofler et al. 2011), including 5 bp above and below each indel, before converting to a sync file and filtering out positions with a base quality score less than 20. Finally, we retained just the autosomes, removing the sex chromosomes, mitochondrial sequences, and other scaffolds. The mean read depth across the genome after filtering was between 22 and 29 depending on the pool.

### 2.3 Statistical Analyses

In the introduction, we introduced our three questions—each of which correspond to specific statistical comparisons for the phenotypic data. (Q1) Phenotypic changes due to the drought were assessed by comparing the pre‐drought samples (2007, 2012, 2013) to the first post‐drought sample (2018) from Rouge Lake. For the three pre‐drought years, sample sizes were low in each year and planned contrasts of interest (2007 vs. 2012; 2012 vs. 2013) did not reveal any significant differences for any of the traits (except for body depth in 2007 to 2012; *p* < 0.05). Hence, we made the decision to pool these three samples into a single pre‐drought sample. (Q2) Phenotypic stability after the recovery was assessed by comparing the first post‐drought sample (2018) to the second post‐drought sample (2022) from Rouge Lake. (Q3) To assess whether the Rouge Outlet population contributed to the Rouge Lake recovery, we compared lake and outlet samples collected in the same year (2022).

#### 2.3.1 Linear Measurements and Meristic Traits

The above three questions involved three different pairwise comparisons between the four samples: Rouge Lake pre‐drought, Rouge Lake post‐drought in 2018, Rouge Lake post‐drought in 2022, and Rouge Outlet post‐drought in 2022 (see above). Hence, the data were analyzed as four levels of one predictor term (‘sample’) in statistical models (see below), followed by planned contrasts between the specific levels of that term that addressed the above questions. In all analyses, sex was included as a fixed factor to account for any sexual dimorphism—and interactions between sex and sample were also considered (details below). Analyses were conducted in R version 4.4.1 (R Core Team 2024).

For linear measurements of each trait (body depth, length of gape, length of the first dorsal spine, length of the second dorsal spine, length of the left pelvic spine, length of the pelvic plate, maximum width of the pelvic plate, height of the ascending process, and maximum width of the ascending process), we used generalized linear models (GLMs) with Gaussian error distributions and an identity link function. We ran a separate GLM for each trait because we are specifically interested in how drought might influence specific traits (see Introduction), as opposed to multivariate trait combinations. The response variable in each case was the allometrically size adjusted and *z*‐transformed trait measurement of each individual. We first fit the models including an interaction between sex and sample (pre‐drought, post‐drought 2018, post‐drought 2022, and outlet 2022). This interaction was never significant, nor did it improve model fit in any case. For these reasons, the final models retained sex and sample but not their interaction. The number of lateral plates was analyzed in similar models but with a Poisson error distribution and a log‐link function. Results were the same if we analyzed lateral plates on the left side of the fish, the right side of the fish, or both sides combined. For simplicity, we only report results for number of plates on the left side.

Two types of post hoc analyses were conducted to compare between traits and samples. First, we used *emmeans* (Lenth 2024) for a priori planned contrast for each of our questions of interest (pre‐drought vs. post‐drought 2018; post‐drought 2018 vs. post‐drought 2022; post‐drought 2022 vs. outlet 2022). We then used the *eff_size* function from the *emmeans* package to calculate standardized effect sizes for each trait. Second, we estimated the partial determination coefficients (partial *R* ^2^) of each GLM predictor by using the *rsq.partial* function of the *rsq* package v2.7 (Zhang 2024). Note that each of these calculations was done separately for each trait for each question to then enable comparisons between them.

#### 2.3.2 Geometric Morphometrics

For the univariate traits described above, it was effective to analyze the sexes together while including sex as a term in the model. Geometric morphometrics, however, are inherently multivariate and calculating the major axes of variation is best done on a sex‐by‐sex basis (Spoljaric and Reimchen 2008) so that sex differences do not confound subsequent analyses. Hence, males and females were analyzed separately for this data type. Analyses were conducted in the *R* program *geomorph* v4.0.8 (Adams et al. 2024; Baken et al. 2021) and *RRPP* v2.0.3 (Collyer and Adams 2018, 2024). We first conducted generalized Procrustes analyses using the *gpagen* function to remove the isometric size effects and achieve uniform orientation and position (Rohlf and Slice 1990). We then conducted a principal component (PC) analysis to identify the major axes of variation among all the fish. For both males and females, PC1 (18% and 23%, respectively) was mainly associated with bending (even after using the *unbend* function in *tpsUtil*). Hence, we focused our analyses on PC2 and PC3. These axes were compared among samples using a Procrustes ANOVA that included centroid size to control for any allometry. We then used the pairwise function from the *RRPP* package to compare the different samples that corresponded to our three questions (see above). Finally, we ran GLMs using PC2 and PC3 as the response variable and centroid size (logged) and sample as fixed effects to replicate the models described above. Using these GLMs, we then conducted planned contrasts as described above.

#### 2.3.3 Genomics

We computed pool‐sequencing corrected genome‐wide measures of genetic diversity in each sample using *Grenedalf* v.0.6.3 (Czech et al. 2024). Specifically, we calculated nucleotide diversity (π), Watterson's estimator (θ_w_), and Tajima's *D* for each pool, filtering out positions with a minimum read count less than 2, a minimum depth less than 4, or a maximum read count greater than 100 (Kofler et al. 2011). Estimates of each metric were made in non‐overlapping 1 kb windows across the genome, averaged across valid loci (i.e., positions that passed the filtering criteria) within each window. We then used block‐jackknife resampling of 50 kb blocks around each window to compute genome‐wide means, standard errors, and 95% confidence intervals for each metric; thus, these represent confidence intervals on variation across the genome. Genome‐wide means were weighted according to the number of valid loci within each block. Finally, we estimated genetic differentiation between the populations as their pair‐wise genome‐wide *F*st using the pool‐sequencing corrected Nei method in *Grenedalf* with the same filtering and block‐jackknife parameters.

## 3 Results

We measured traits on a total of 252 fish: 45 from the pre‐drought sample (3 years combined), 88 from the post‐drought 2018 sample, 99 from the post‐drought 2022 sample, and 20 from the outlet 2022 sample (Table 1). The average fish size (standard length) across all samples was 51.90 mm ±8.65 (SD). Both pre‐drought and post‐drought pH levels remained very low in the lake, 4.1–4.5 and 4.6, respectively.

### 3.1 Q1: To What Extent Have Phenotypic Traits Changed From Before to After the Drought?

**FIGURE 3:**
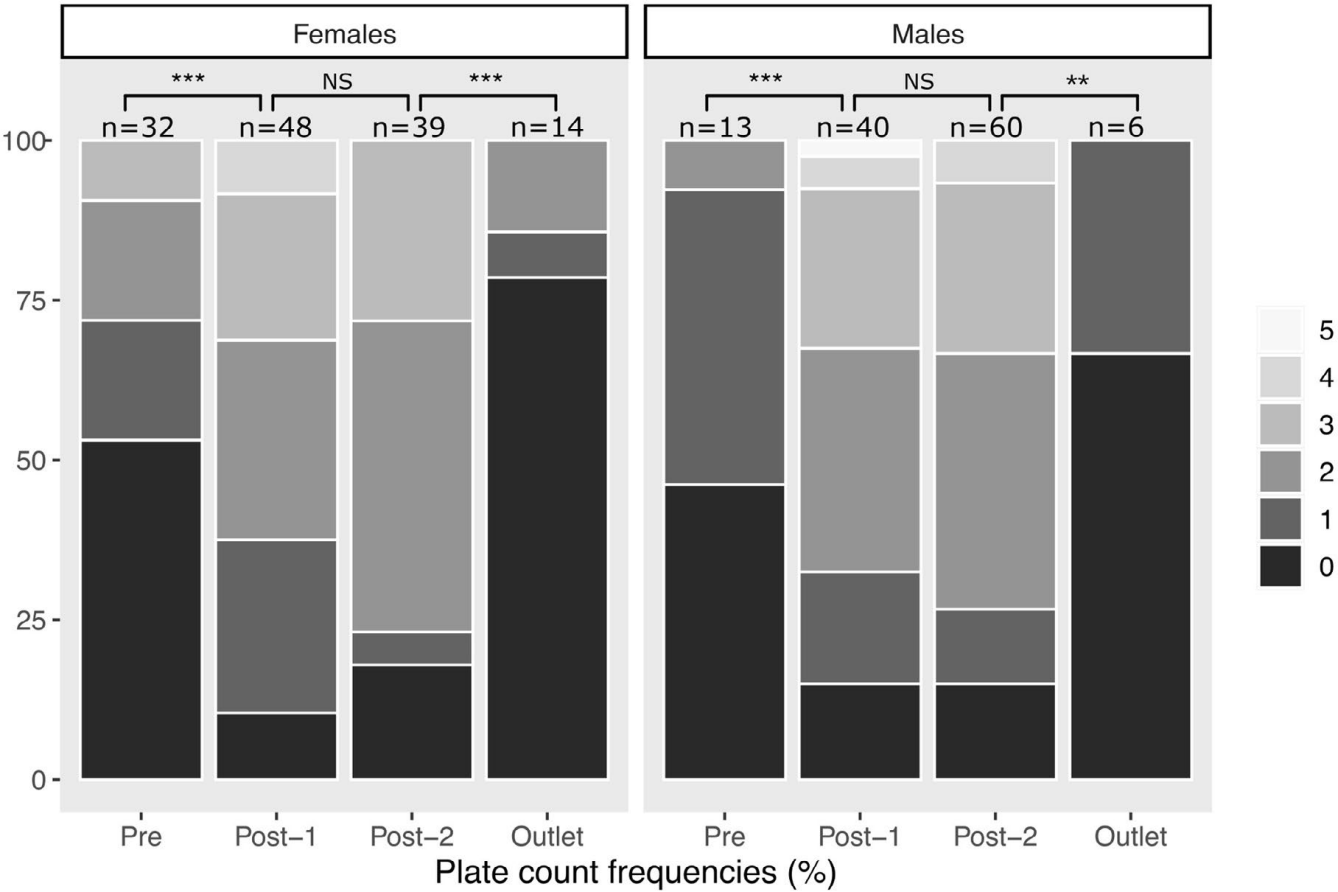
Frequencies of lateral plate counts (left side) for fish from the different samples (lake ‘Pre’‐drought, lake ‘Post‐1’ drought 2018, lake ‘Post‐2’ drought 2022, and ‘Outlet’ 2022). Left panels are females and right panel are males. The *x*‐axis shows frequencies of each count as different shades of gray. Statistical significance between samples are indicated by brackets: ***p < 0.001, **p < 0.01. The *p*‐values are extracted from a GLM with sex and sample as predictor variables. Statistical significance comparisons correspond to the questions outlined in the introduction: Q1: Pre‐drought vs. Post‐drought 1 (2018), Q2: Post‐drought 1 (2018) versus Post‐drought 2 (2022), and Q3: Post‐drought 2 (2022) versus Outlet (2022).

Considering the original unarmoured status of this population, by far the most striking difference between the pre‐drought sample and the first post‐drought sample (2018) was in the number of lateral plates (*p* < 0.001, *R* ^2^ = 0.20, *d* = −0.91). In the pre‐drought sample, 51% of the fish had no lateral plates on the left side, whereas in the post‐drought sample of 2018, only 13% had no lateral plates on the left side. Stickleback from the pre‐drought sample had an average total number of lateral plates of 1.6, increasing to 3.7 after the drought. These increases were consistent in both females and males (females: 1.6 to 3.4; males: 1.6 to 4.0), although females had consistently fewer plates than males overall (Figure 3). The increase in lateral plate number was most often in positions 5, 6, 7 (Table S1), which are the plates that buttress the basal plates of the dorsal spines (Reimchen 1983).

**FIGURE 4:**
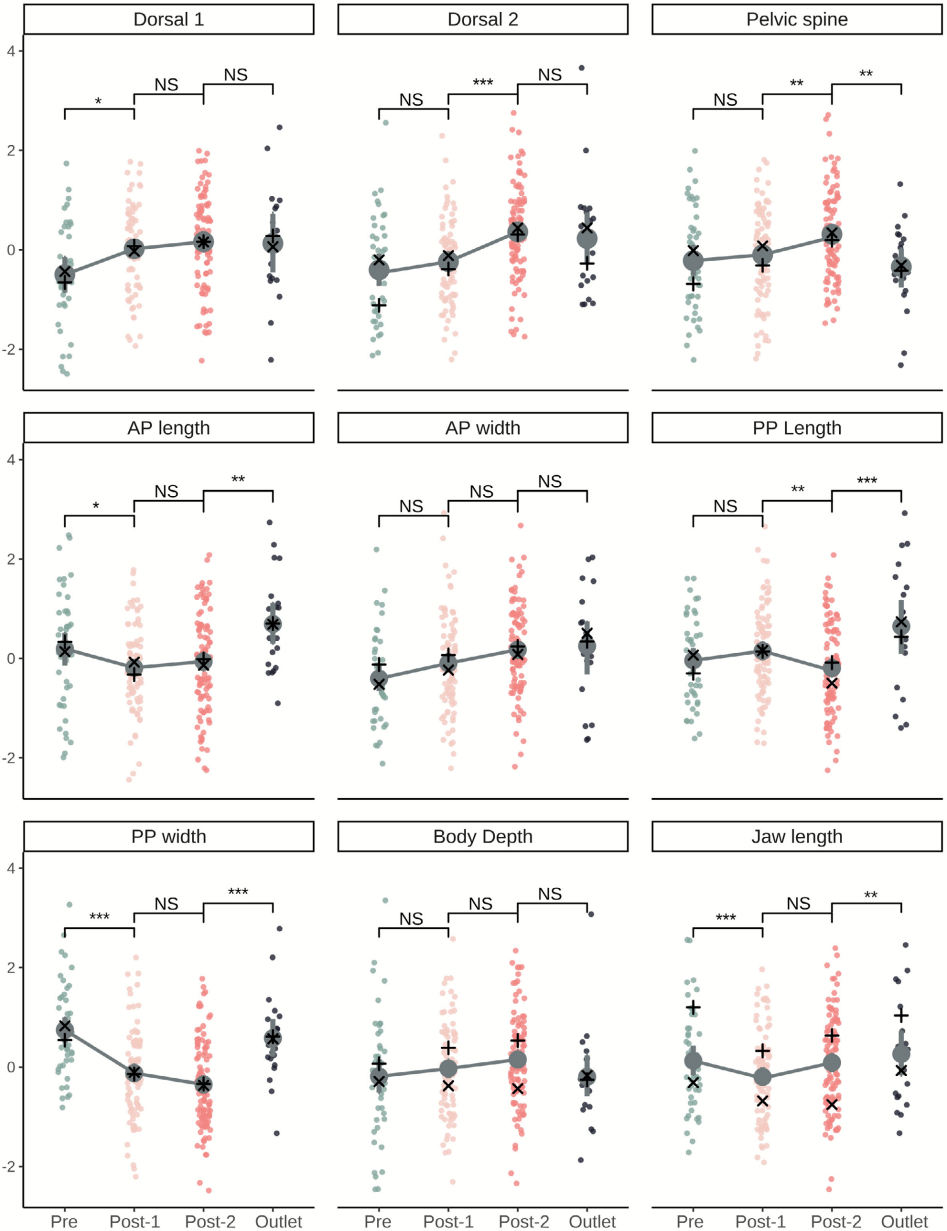
Allometrically size adjusted and *z*‐transformed trait measures per sample. Error bars are 95% bootstrapped confidence intervals for the sample means. Statistical significance between samples is indicated by brackets: ***p < 0.001, **p < 0.01, *p < 0.05. These *p*‐values are extracted from GLMs with sex and sample as predictor variables. Only comparisons of interest are depicted: Pre‐drought versus Post‐drought 2018, Post‐drought 2018 versus Post‐drought 2022, and Post‐drought 2022 versus Outlet. Trait means for females are depicted with x and means for males with *+*. Dorsal spine 1 was absent in some populations (not included in figure): Pre‐drought: *n* = 5; post‐drought 1: *n* = 6, post‐drought 2: *n* = 8; outlet: *n* = 3.

The other traits showed weaker and more variable patterns that were similar when the sexes were analyzed together (Figure 4) or separately (see Figures S2 and S3). Here we summarize the changes that were statistically significant, while the complete results for all traits are reported in Table 2, Table S2, and Figure 4. First, the length of the first dorsal spine increased from before to after the drought, although the change was small (*p* = 0.011, *R* ^2^ = 0.06, *d* = −0.45). Second, several other defensive traits decreased somewhat in size, including pelvic plate width (*p* < 0.001, *R* ^2^ = 0.16, *d* = 0.94) and ascending process length (*p* = 0.036, *R* ^2^ = 0.03, *d* = 0.39). Finally, jaw length decreased from before to after the drought (*p* < 0.001, *R* ^2^ = 0.10, *d* = 0.71), although sex explained much more of the variation for this trait (*R* ^2^ = 0.34).

**TABLE 2:** Results from generalized linear models where trait is the response variable and sex and sample (pre‐drought (2007–2012, 2013), post‐drought 2018, post‐drought 2022, and outlet (2022)) are predictor variables.

	Estimate	Std error	*t* value	*p*
Body depth				
Intercept (F‐pre)	−0.396	0.144	−2.758	**0.006**
Sex (M)	0.721	0.122	5.920	**< 0.001**
2018	0.039	0.172	0.227	0.821
2022	0.111	0.172	0.643	0.521
Outlet	−0.017	0.251	−0.067	0.947
AP length				
Intercept (F‐pre)	0.197	0.150	1.312	0.191
Sex (M)	−0.011	0.127	−0.084	0.933
2018	−0.380	0.180	−2.103	**0.036**
2022	−0.252	0.180	−1.399	0.163
Outlet	0.498	0.263	1.893	0.059
AP width				
Intercept (F‐pre)	−0.470	0.149	−3.147	**0.002**
Sex (M)	0.226	0.126	1.791	0.075
2018	0.270	0.179	1.510	0.132
2022	0.509	0.179	2.845	**0.005**
Outlet	0.859	0.261	3.295	**0.001**
Jaw length				
Intercept (F‐pre)	−0.235	0.121	−1.944	**0.053**
Sex (M)	1.247	0.102	12.199	< 0.001
2018	−0.556	0.145	−3.839	**< 0.001**
2022	−0.433	0.145	−2.991	**0.003**
Outlet	0.123	0.211	0.585	0.559
PP length				
Intercept (F‐pre)	−0.063	0.150	−0.423	0.672
Sex (M)	0.081	0.127	0.640	0.523
2018	0.177	0.180	0.984	0.326
2022	−0.232	0.180	−1.290	0.198
Outlet	0.685	0.262	2.618	**0.009**
PP width				
Intercept (F‐pre)	0.758	0.140	5.401	**< 0.001**
Sex (M)	−0.043	0.119	−0.358	0.721
2018	−0.861	0.168	−5.112	**< 0.001**
2022	−1.082	0.169	−6.415	**< 0.001**
Outlet	−0.164	0.245	−0.668	0.505
Dorsal spine 1				
Intercept (F‐pre)	−0.505	0.168	−3.004	**0.003**
Sex (M)	0.026	0.137	0.189	0.851
2018	0.512	0.199	2.575	**0.011**
2022	0.651	0.200	3.253	**0.001**
Outlet	0.626	0.295	2.121	**0.035**
Dorsal spine 2				
Intercept (F‐pre)	−0.366	0.148	−2.465	**0.014**
Sex (M)	−0.343	0.123	−2.794	**0.006**
2018	0.282	0.177	1.595	0.112
2022	0.934	0.177	5.289	**< 0.001**
Outlet	0.696	0.254	2.739	**0.007**
Pelvic spine				
Intercept (F‐pre)	−0.123	0.153	−0.802	0.423
Sex (M)	−0.317	0.127	−2.498	**0.013**
2018	0.168	0.182	0.924	0.356
2022	0.567	0.182	3.121	**0.002**
Outlet	−0.125	0.263	−0.474	0.636
Lateral plates			*z* value	
Intercept (F‐pre)	−0.255	0.712	−1.485	0.128
Sex (M)	0.012	0.102	0.117	0.907
2018	0.907	0.186	4.871	**< 0.001**
2022	0.910	0.187	4.876	**< 0.001**
Outlet	−0.801	0.414	−1.929	**0.054**

*Note:* Significance values < 0.05 are given in bold.

**FIGURE 5:**
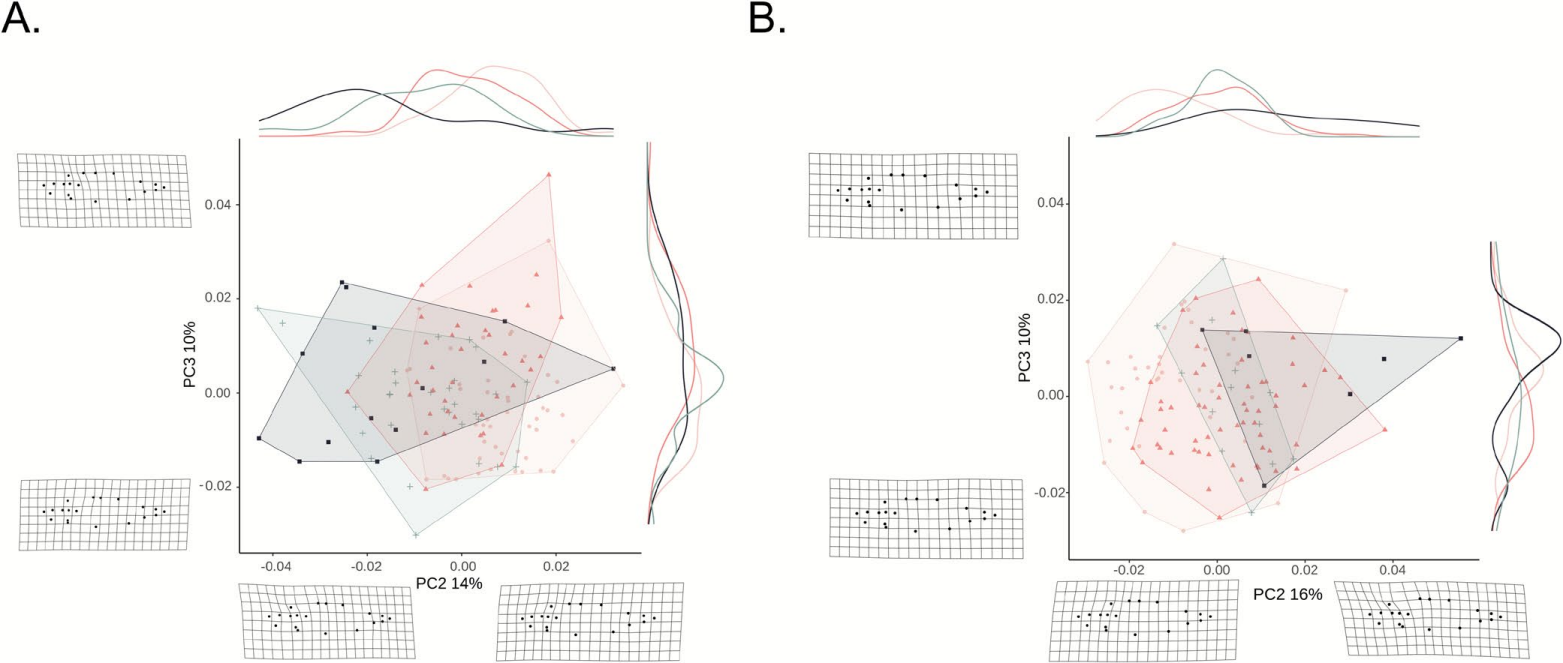
Morphospace of female stickleback (Panel A) and male stickleback (Panel B) from PCA of landmark data, with PC2 on the horizontal axis and PC3 on the vertical axis. PC1 is not illustrated as it was closely related to bending associated with placement of fish for photographs. Green crosses are Pre‐drought (2007, 2012, 2013), light pink dots are fish from the lake Post‐drought 2018, dark pink triangle are fish from the lake Post‐drought (2022), and black squares are fish from the Outlet (2022). Relative frequency densities for each PC are shown along the PC axes. Deformation grids illustrating extreme PC value for each axis.

For female geometric morphometrics, PC2 explained 14% of the variation and PC3 explained 10% of the variation. Only PC2 differed between the pre‐drought sample and the post‐drought 2018 sample (*p* < 0.0001). Specifically, females from the pre‐drought sample have the tip of the supraoccipital crest positioned closer to the tip of the pre‐maxilla than do the post‐drought females (Figure 5). For male geometric morphometrics, PC2 explained 16% of the variation and PC3 explained 10% of the variation. As in females, the pre‐drought males have the tip of the supraoccipital crest positioned closer to the tip of the pre‐maxilla than the post‐drought males (*p* = 0.001). For both sexes, this change in the position of the supraoccipital crest is suggestive of a loss in the postcranial hump (Figure 5). PC3 did not differ between the pre‐drought and post‐drought sample (*p* = 0.37). Irrespective of statistical significance, however, we note that the differences between samples were quite small in comparison to the variation within them (Table 3, i.e., females: *R* ^2^ = 0.11–0.79; males: *R* ^2^ = 0.11–0.80).

**TABLE 3:** Summary of Procrustes ANOVA describing the relationship between body shape of stickleback and predictors, including centroid size (a proxy for fish size calculated from two‐dimensional shape data) and sample (pre‐drought, post‐drought 2018, post‐drought 2022, outlet).

Term	df	SS	MS	*R* ^2^	*F*	*Z*	*p*
Females							
Centroid size	1	0.015	0.015	0.079	12.548	6.532	**< 0.001**
Sample	3	0.021	0.007	0.111	5.923	6.701	**< 0.001**
Residuals	126	0.152	0.001	0.790			
Total	130	0.193					
Males							
Centroid size	1	0.013	0.013	0.084	12.040	6.322	**< 0.001**
Sample	3	0.018	0.006	0.111	5.288	7.549	**< 0.001**
Residuals	114	0.126	0.001	0.800			
Total	118	0.157					

*Note:* Significant *p*‐values (< 0.05) are bolded.

### 3.2 Q2: Are Trait Changes Post‐Drought Stable During the Recovery?

The substantial increase in lateral plates from before to after the drought (Q1—above) remained stable for at least the following 4 years (*p* = 0.984, *R* ^2^ = < 0.001, *d* = −0.002). For instance, the percentage of fish with no lateral plates on the left side was 13% in 2018 and 16% in 2022. The total number of lateral plates averaged 3.7 in 2018 and 3.6 in 2022, without any differences between the sexes (Table 2; Figure 3).

Most of the other linear traits also did not show any change between the first (2018) and second (2022) post‐drought samples—with three exceptions. Specifically, modest increases were seen in the length of the second dorsal spine (*p* < 0.001, *R* ^2^ = 0.11, *d* = −0.70) and the pelvic spine (*p* = 0.006, *R* ^2^ = 0.04, *d* = −0.41). A modest decrease was seen in the length of the pelvic plate (*p* = 0.005, *R* ^2^ = 0.05, *d* = 0.42). For both the second dorsal spine and the pelvic spine, the increase was not in the direction of the initial pre‐drought population (Figure 4). The decrease in pelvic plate length, however, was in the direction of the initial pre‐drought population, becoming even shorter than in the pre‐drought sample (Figure 4).

For female geometric morphometrics, both PC2 and PC3 were different between the two post‐drought samples (*p* < 0.008 and *p* = 0.0002). In general, these results reflected the tip of the supraoccipital crest moving back closer to the tip of the pre‐maxilla in 2022 and the insertion of the first dorsal spine moving slightly closer to the tip of the pre‐maxilla in 2022. For males, both axes of variation (PC2 and PC3) were also different between the two post‐drought samples (*p* < 0.0001 and *p* = 0.01), and the trait shifts were similar to females. Importantly, however, all of the differences between samples were very small (Figure 5).

### 3.3 Q3: Did the Population Recover in Situ or via Immigration From Rouge Outlet?

In 2022, most traits showed a difference between the lake and the outlet (Figure 4; Table 2; Table S2). For instance, the number of fish with no lateral plates was 16% in the lake and 75% in the outlet (Figure 3). As another example from geometric morphometric results, females in the lake had first dorsal spine insertions closer to the head compared to females from the outlet (Figure 5). Of particular interest, outlet females were more similar to lake females before the drought than they were after the drought for 8 out of 11 traits.

Rouge Lake and Rouge Outlet showed low differentiation based on a genome‐wide estimate of *F*st = 0.0237 (95% CI: 0.0232–0.0242). This degree of differentiation was similar (Table 4) to that between each other lake (Drizzle and Mayer) and their corresponding experimental pond (*F*st = 0.0228 for Drizzle; *F*st = 0.0340 for Mayer). At the same time, these non‐Rouge Lake samples are very different from Rouge Lake (*F*st = 0.3456–0.2941) and the Rouge Outlet (*F*st = 0.3139–0.2662).

**TABLE 4:** Pairwise genome‐wide *F*st estimates between all sequenced populations with 95% confidence. The comparison between Rouge Lake and Outlet is highlighted in grey, as are the comparisons between experimental lake–pond pairs (see text for details).

	Rouge Lake	Rouge Outlet	Drizzle Lake	Drizzle Pond	Mayer Lake	Roadside Pond
Rouge Lake	—					
Rouge Outlet	0.0237 ± 0.0005	—				
Drizzle Lake	0.3456 ± 0.0038	0.3139 ± 0.0037	—			
Drizzle Pond	0.3313 ± 0.0036	0.3005 ± 0.0034	0.0228 ± 0.0004	—		
Mayer Lake	0.2941 ± 0.0032	0.2663 ± 0.0031	0.143 ± 0.0022	0.1248 ± 0.0018	—	
Roadside Pond	0.2980 ± 0.0030	0.2693 ± 0.0029	0.1522 ± 0.0002	0.1312 ± 0.0017	0.0340 ± 0.0008	—

In comparison to all of the sequenced non‐Rouge populations (including the experimental ponds), both Rouge Lake and Rouge Outlet had the lowest nucleotide diversity (Table 5; Lake π = 0.0033, 95% CI = 0.0032–0.0034; Outlet π = 0.0037, 95% CI = 0.0037–0.0038) and the most negative values of Tajima's *D* (Rouge *D* = −3.5497, 95% CI = −3.5681 to −3.5314; Outlet *D* = −3.4515, 95% CI = −3.4698 to −3.4332). These estimates were significantly lower than those from both the lakes and introduction ponds in all comparisons (Welch's *t*‐test: *p* < 0.001 in all cases). Tajima's *D* is generally expected to increase (become more positive) immediately following a bottleneck but then trend more negative over subsequent generations (Gattepaille et al. 2013). However, Tajima's *D* is not well‐suited to several aspects of pool‐sequencing data (Czech et al. 2024) and is more likely to reflect long‐term demographic histories rather than very recent events, making it hard to interpret without an estimate from before the drought (Clark et al. 2024). For example, in the two experimental pond introductions in which bottlenecks occurred many generations earlier than the drought in Rouge Lake, one shows a negative trend in Tajima's *D* between the source lake and experimental pond (Mayer Lake to Roadside Pond), whereas the other shows a positive trend (Drizzle Lake to Drizzle Pond). This finding highlights that diversity‐based metrics like Tajima's *D* might have limited utility for inferring recent demographic histories, although datasets with multiple timepoints might help to overcome these challenges. Future work could combine such datasets with empirically parameterised simulations of multiple evolutionary scenarios, providing estimates of how these metrics should change (and confidence intervals around those changes) according to shifts in demography and selection.

**TABLE 5:** Estimates of genetic diversity in each of the sequenced populations with 95% confidence intervals.

	Rouge Lake	Rouge Outlet	Drizzle Lake	Drizzle Pond	Mayer Lake	Roadside Pond
Nucleotide Diversity (π)	0.0033 ± 0.0001	0.0037 ± 0.0001	0.0050 ± 0.0001	0.0052 ± 0.0001	0.0059 ± 0.0001	0.0058 ± 0.0001
Segregating Sites (θ_w_)	0.0125 ± 0.0002	0.0131 ± 0.0002	0.0135 ± 0.0003	0.0135 ± 0.0003	0.0119 ± 0.0002	0.0127 ± 0.0002
Tajima's *D*	−3.5497 ± 0.0 184	−3.4515 ± 0.0183	−2.9438 ± 0.0204	−2.8524 ± 0.0195	−2.1176 ± 0.0196	−2.3499 ± 0.0200

## 4 Discussion

Our work focused on how a severe drought influenced the Unarmoured Stickleback population in Rouge Lake (Haida Gwaii, BC, Canada) that is currently listed as Special Concern under the Canadian Species at Risk Act (SARA) and Endangered by COSEWIC. In addition to a severe population decline (see Introduction) and a corresponding demographic bottleneck (details below), the population showed substantial phenotypic changes. Interestingly, these changes were largest in the ‘unarmoured’ phenotype that was a core motivation for their initial listing. Specifically, 51% of the fish had no lateral plates before the drought, whereas only 13% of the fish had no lateral plates after the drought (Figure 3). This shift in plate number then seemingly persisted until at least 4 years later, well after water levels had returned to their original state. Several other traits also changed following the drought; although those shifts were not as consistent or dramatic (Figure 4; Table 2; Table S2), we will explain how they could be informative regarding potential shifts in the environment.

In the following sections, we discuss findings related to our three questions by leveraging the detailed ecological, functional, and genetic information available for stickleback generally and the Rouge Lake population more specifically. Afterwards, we consider future research directions and implications for conservation and management.

### 4.1 Q1: To What Extent Have Phenotypic Traits Changed From Before to After the Drought?

Although the change in lateral plates from before to after the drought was substantial (Figure 3), we note that rapid changes in lateral plate numbers have been documented in other instances of stickleback populations experiencing environmental change—most obviously when anadromous populations colonize new freshwater sites (Barrett et al. 2008; Francis et al. 1985; Hagen and Gilbertson 1972). As a specific example, Loberg Lake, AK, USA was poisoned with rotenone in 1982 (Bell 2001; Bell et al. 2004). In 1990, anadromous stickleback became established with 96% being completely plated. By 1993, only 40% were completely plated. As the gene (EDA) underlying ‘plate morph’ controls a large proportion of plate number variation, the rapid change was likely genetic (Colosimo et al. 2004, 2005)—providing one of the most dramatic examples of rapid evolution (Bell et al. 2004). Moreover, Barrett et al. (2008) showed that these rapid changes in plate morphs can occur within a single generation when marine stickleback are introduced into freshwater. These rapid changes in the low‐plate allele frequency are likely due to a growth advantage in freshwater (Barrett et al. 2008). However, all of the fish in Rouge Lake are already of the ‘low‐plate’ morph, and so more direct comparisons would be changes in plate number within strictly freshwater populations—and we can here point to two examples from Haida Gwaii. First, mean lateral plate number in subadults in Drizzle Lake increases by about one lateral plate per side during the winter and spring (when fish predation is relatively high) and decreases by the same amount in the summer and fall (when bird predation is greatest) (Reimchen 1995). Second, when stickleback from Mayer Lake were introduced into a fishless Roadside Pond, plate number decreased from 7.4 to 6.4 plates (per side) over seven generations, a change suggested to be associated with the lack of vertebrate predators in the lake (Leaver and Reimchen 2012; Marques et al. 2018). These changes in lateral plate number likely reflect genetic changes in modifier QTLs that are unlinked to EDA and cause variation in plate number within the low‐plate morph category (Barrett et al. 2008; Colosimo et al. 2004; Richmond et al. 2015).

These drought‐associated changes in lateral plate number in Rouge Lake could have been induced by strong directional selection, genetic drift, new founders, or a combination of these processes. Because our data cannot confidently distinguish between these possibilities, we discuss each mechanism below. The remainder of this section therefore outlines scenarios for how selection and/or genetic drift might have contributed to the observed increase in lateral plate number, while Q3 later discusses the possibility of new founders colonizing the lake.

Changes in lateral plate number in stickleback often reflect selection associated with changes in predation; but was this the case for Rouge Lake? The presence of predatory fish generally selects for more numerous lateral plates (Haines et al. 2025; Kitano et al. 2008; Reimchen 1983, 1992, 1994), whereas the presence of piscivorous birds (Reimchen 1980, 1983) and macro‐invertebrate predators (Foster et al. 1988) generally selects for fewer lateral plates. Thus, if adaptive, the increase in lateral plates in Rouge Lake could suggest a shift in the selective landscape from bird and invertebrate dominated predation to fish dominated predation. Before the drought, Rouge Lake was known to contain small numbers of *Dolly* Varden ( *Salvelinus malma* , Walbaum 1792) and occasional juvenile coho ( *Oncorhynchus kisutch* , Walbaum 1792) (Reimchen and Buckland‐Nicks 1990), dytiscid larva, and Red‐throated Loons ( *Gavia stellata* , Pontoppidan 1763), all of which consume stickleback. We suggest that decreasing water levels could have increased susceptibility to resident predatory salmonids while also decreasing the presence of birds and invertebrate predators. As we did not collect data on predators during the drought, we cannot be definitive on these possibilities. However, we also note that the increase in lateral plates observed in Rouge Lake was mainly associated with plates in positions 5 through 7 (Table S2), which are particularly important—during predation events—in supporting the dorsal spines (Reimchen 1983), which also saw an increase in size (Figure 4). It does seem unlikely that salmonids survived the lowest water levels; indeed, we did not see them in the remaining puddle when visiting in 2015. Instead, any selection they imposed would likely have taken place earlier in the drought when water levels had decreased but not to the most extreme low level.

Beyond the dramatic change in lateral plates, we recorded several additional phenotypic trait changes following the drought (Table 2; Figure 4; Table S2), with the largest changes seen in jaw length and pelvic plate length (Figure 4; Table S2). Both types of trait changes are known to occur when stickleback encounter new environments (Leaver and Reimchen 2012; Schluter and McPhail 1992). For instance, relatively shorter gapes are normally associated with foraging on zooplankton, whereas longer gape length and width are typically associated with foraging on benthic macroinvertebrates (Schluter 1993, 1995; Schluter and McPhail 1992). Thus, the trait shifts we observed point towards a shift in stickleback diet after the drought to an increase in zooplankton, which might have recovered faster than macroinvertebrates. In addition, our geometric morphometric results suggest a decreasing size of the postcranial hump in both males and females, a trait which was rather extreme in the pre‐drought population (Reimchen 1984; Figure 5). A more ‘humped’ phenotype is generated by hypertrophied epaxial muscles which are typical of benthic fish (McPhail 1992)—and so the observed decrease in hump size also suggests a decrease in benthic foraging.

During the extreme low water period itself, the population clearly underwent a strong demographic bottleneck (supported by field observation and subsequent sampling). Extreme bottlenecks of this type reduce effective population size, and thus can amplify stochastic changes in allele frequencies, while also causing the loss of rare alleles, increased homozygosity, and higher fixation rates (Nei et al. 1975). Even for polygenic traits, bottlenecks can move the mean phenotype away from the optimum (Stephan and John 2020). As such, we cannot rule out the possibility of this demographic bottleneck contributing to the observed increase in lateral plate number in Rouge Lake. However, regardless of the mechanisms responsible for the changes in Rouge Lake, the key point holds: an extreme environmental disturbance caused a severe reduction in population size and a loss of a unique feature in this population.

As noted above, the observed changes in lateral plates were very likely to be genetic. Changes in the other traits probably had a larger plastic contribution. Common‐garden studies show that many trait differences are weaker after removing environmental differences (Oke et al. 2016). In addition, the time course of trait changes in experimental introductions, such as the aforementioned Mayer Lake to Roadside Pond introductions (Spoljaric and Reimchen 2007), often show a large immediate jump (probably reflecting plasticity) followed by much slower subsequent change (probably reflecting genetic change). In short, we suspect that much of the change in traits other than plate number is the result of phenotypic plasticity, or, at least, we have no way of excluding that possibility.

### 4.2 Q2: Are Trait Changes Post‐Drought Stable During the Recovery?

For nearly all of the traits we examined, including lateral plates, the shifts observed from before to after the drought did not later revert back to the pre‐drought phenotype in subsequent years (Figure 4). In some cases, including for lateral plates, no additional change was seen after the initial drought‐associated shift: that is, the change that took place during the drought was seemingly stable afterwards. However, changes in lateral plates can occur rapidly (Barrett et al. 2008; Leaver and Reimchen 2012; Reimchen 1995) and it remains possible that changes occurred during our four‐year sampling period. In other cases, such as dorsal and pelvic spine lengths, changes seen during the drought seem to continue in the same direction in subsequent years (Figure 4). In only one case, pelvic plate length, did the shift seen during the drought show a clear subsequent trend back towards the pre‐drought state (Figure 4). As another potential case, the jaw length increase during the drought might be reverting—but only for males and without statistical significance (Figure S3). Even in this last case, however, the post‐drought shift from 2018 to 2022 returned the population only 36% of the way back to the pre‐drought state.

When a disturbance leads to abrupt evolutionary shifts that are not quickly reversed after the disturbance is gone, several explanations are possible. First, the selective environment, such as the predation regime (as discussed above), might have shifted. Second, the selective regime might have reverted back to the original state, but ‘reverse evolution’ might be slow due to various constraints. For instance, strong demographic bottlenecks could reduce the genetic variation necessary for reverse evolution (Porter and Crandall 2003), or ongoing gene flow from another population could be counteracting selection (Hendry et al. 2001). These constraints seem unlikely in the present case because the core trait of interest still shows considerable variation (16% of the fish remain unarmoured after the drought) and gene flow seems unlikely to have changed—at least not permanently (see below). Alternatively, it might take more time for the population to return to its original phenotype because selection is now ‘relaxed’ (Lahti et al. 2009). An example can be found in laboratory experiments on Atlantic silversides ( *Menidia menidia* , Linnaeus 1766) where size‐selective harvest treatments lead to rapid evolution, whereas the cessation of harvest treatments led to much slower reverse evolution (Conover et al. 2009).

### 4.3 Q3: Did the Population Recover in Situ or via Immigration From Rouge Outlet?

Two main scenarios could explain post‐drought recovery of the Rouge Lake population. First, the population could have gone locally extinct during the drought and then been subsequently recolonized by fish from the nearby Rouge Outlet, the only logical other source given the absence of an inlet population. Alternatively, the lake population might have gone through a massive demographic bottleneck without going extinct, with the few fish surviving the drought in situ then driving subsequent recovery. As genomic data were only available after the drought, we cannot confidently distinguish between the two recovery scenarios. Specifically, the two populations were genetically different but only to a small degree (*F*st = 0.0237, 95% CI = 0.0232–0.0242), which could be consistent with either scenario. On the one hand, the small genetic difference could have arisen due to a founder effect (if only a few outlet fish recolonized the lake) perhaps with evolutionary shifts afterwards. On the other hand, the only small genetic difference between the lake and outlet could simply reflect gene flow from the lake to the outlet (the reverse is unlikely—see below)—as the outlet sample was from very near (150 m) the lake. Indeed, other lake and outlet populations show similarly small genetic differences on such scales (Berner et al. 2009; Bolnick et al. 2009).

Considering all of the available data and information, we favour the in situ recovery scenario, although we cannot rule out the possibility of outlet fish contributing to the recovery. Our morphological results suggest that the outlet fish did not recolonize the lake after the drought (Tables 2 and 3; Figures 3, 4, 5; Table S2). The reason is that outlet fish resemble pre‐drought fish much more closely than post‐drought fish for the majority of traits—especially lateral plates (Figure 3, Table S2). However, we cannot be sure of this interpretation because stickleback found further down the outlet (closer to the ocean) tend to be more plated and could potentially have travelled up the outlet to recolonize the lake (Deagle et al. 1996). Although stickleback can travel considerable distances, previous genetic work in this system suggests some downstream gene flow from the lake to the outlet, but no upstream gene flow from the outlet to the lake (Deagle et al. 1996). In addition, beaver activity in the 1970s increased water levels in the lake and simultaneously created a barrier to upstream movement of stream fish (Reimchen 1984). Finally, the stream population would also have been severely impacted by the drought (including reduced movement).

Regardless of the colonization scenario, however, substantial phenotypic change clearly took place during the drought. That is, the post‐drought lake population is phenotypically very distinct from the pre‐drought lake population and the outlet population—both in our 2022 samples and as reported in earlier studies (Deagle et al. 1996; Reimchen 1984). We hope that future application of ancient DNA protocols will enable pre‐drought genomic inferences that could provide more definitive answers.

### 4.4 Implications for Conservation Strategies

The substantial and persistent change in the number of lateral plates in Rouge Lake stickleback from before to after the drought raises questions for conservation strategies and mitigation. Endemic and distinct populations like the Unarmoured Stickleback in Rouge Lake are often protected under SARA (or ESA in the United States) because they are both discrete and evolutionarily significant. However, when these populations evolve rapidly to (at least partially) lose some of their unique features because of environmental disturbances, as appears to be the case for the Rouge Lake stickleback, their conservation status might be called into question. That is, some might argue that the current population of stickleback in Rouge Lake has lost its evolutionary distinctiveness, such that it no longer constitutes a Designatable Unit and therefore no longer warrants special protection. This scenario is not simply academic. It has happened previously with the same species in the same Federal and Provincial jurisdictions (although both governments have now recognised Haida land title). For example, the collapse of the Enos Lake species pair (see the Introduction) has led to the COSEWIC recommendation of an *Extinct* designation (COSEWIC 2023a, 2023b, 2023c).

In cases such as the one we have described, we can envision three possible conservation management approaches. First, managers might consider a conservation translocation. For instance, other Unarmoured Stickleback populations (also listed under SARA) exist in Haida Gwaii: specifically, Boulton Lake and Serendipity Lake (Reimchen 1980, 1984). In theory, these populations could be used to artificially recolonize Rouge Lake. However, the different unarmoured populations (Rouge, Boulton, and Serendipity) are evolutionarily independent and show considerable differences in other phenotypic traits (Reimchen 1980, 1984). As such, this first strategy seems inadvisable. Second, the Unarmoured Stickleback from Rouge Lake could potentially be demoted by COSEWIC, as was the case for Enos Lake. However, the persistence of un‐plated individuals in Rouge Lake suggests that the genetic variation necessary for evolution back to a mostly unarmoured state remains a possibility. This realization points towards the third approach—retaining the current protection level and monitoring the population to see if it evolves back to its original state. We strongly recommend this last approach as the variation in armour remains present and the population is unique in other respects that we have detailed above.

Conservation strategies often rely upon static evolutionary baselines, yet our results underscore the need for more dynamic approaches when protecting and monitoring listed populations. As anthropogenic stressors and global climate change continue to drive rapid evolutionary changes (Hendry and Kinnison 1999; Alberti et al. 2017; Sanderson et al. 2021), it has become increasingly clear that conservation management must incorporate such dynamics (Cook et al. 2021; Thurman et al. 2022; Thompson et al. 2023). Threespine stickleback, for instance, are renowned for their ability to rapidly adapt to diverse habitats. Although this adaptability defines them as an evolutionary model species (Bell and Foster 1994), it also renders them prone to evolutionary change when their environments shift, even slightly. In such cases, key traits that initially justified a population's conservation status can change substantially within just a few generations. As another example, male stickleback in Rouge Lake used to have bright red nuptial colouration but lost this trait following the introduction of beavers that subsequently increased water levels (Reimchen 1984). If the goal of conservation management in Canada is to protect discreetness and evolutionary significance, our findings highlight the need for more monitoring to better assess ongoing evolutionary changes.

In summary, our study highlights how extreme environmental events can have profound impacts on evolutionarily distinct populations. Not only are these populations at risk of extinction, but they can also be at risk of losing the very characteristics that motivated their conservation status and made them unique. As a result, even if these populations continue to persist, they might no longer represent evolutionarily significant or discrete populations. Our suggestion in such cases is to focus on whether the original variants are still present, thus allowing the potential for evolution back to the original ‘unique’ state. Similar conclusions have been advanced for other species, such as Pacific salmon with distinct spawning times that have mostly disappeared and yet retain the allele that could allow re‐evolution of the original type (Oke and Hendry 2019; Thompson et al. 2019).

## Funding

This work was supported by Canada Research Chairs, the Natural Sciences and Engineering Research Council of Canada (569441, NRC2354) and the Québec Centre for Biodiversity Sciences (QCBS).

## Conflicts of Interest

The authors declare no conflicts of interest.

## Supporting information


**Table S1:** Percentage of individuals per sample with most common anterior lateral plate position (left side) phenotypes (see Figure 2; panel A for depiction of plate position). Plate no. refers to the total number of lateral plates present (left side only) and plate position refers to the position where the plates are present (e.g., “5,6,7” means that fish have lateral plates present in positions 5, 6, and 7). Pre‐drought samples combine fish collected in years 2007, 2012, and 2013.
**Table S2:** Planned contrasts (pre‐drought vs. post‐drought 2018; post‐drought 2018 vs. post‐drought 2022; post‐drought 2022 vs. outlet) from GLMs where trait is the repones variable, and sex and sample are the fixed effects.
**Figure S1:** Timeline depicting sample sizes (above) per year (below) and samples used for whole‐genome pool‐sequencing.
**Figure S2:** Allometrically size adjusted and z‐transformed trait measures per sample for females only. Error bar are 95% bootstrapped confidence intervals for the sample means. Statistical significance between samples is indicated by brackets: *** *p* < 0.001, ** *p* < 0.01, *p < 0.05. These *p*‐values are extracted from GLMs with sex and sample as predictor variables. Only comparisons of interest are depicted: Pre‐drought versus Post‐drought 2018, Post‐drought 2018 versus Post‐drought 2022 and Post‐drought 2022 versus Outlet. Dorsal spine 1 was absent in some populations (not included in figure): Pre‐drought: *n* = 4; Post‐drought 2018: *n* = 4, Post‐drought 2022: *n* = 4; Outlet: *n* = 3.
**Figure S3:** Allometrically size adjusted and z‐transformed trait measures per sample for males only. Error bar are 95% bootstrapped confidence intervals for the sample means. Statistical significance between samples is indicated by brackets: *** *p* < 0.001, ** *p* < 0.01, *p < 0.05. These *p*‐values are extracted from GLMs with sex and sample as predictor variables. Only comparisons of interest are depicted: Pre‐drought versus Post‐drought 2018, Post‐drought 2018 versus Post‐drought 2022 and Post‐drought 2022 versus Outlet. Dorsal spine 1 was absent in some populations (not included in figure): Pre‐drought: *n* = 1; Post‐drought 2018: *n* = 2, Post‐drought 2022: *n* = 4; Outlet: *n* = 0.

## Data Availability

Data for this study are available at Dryad with DOI: https://doi.org/10.5061/dryad.prr4xgz1m.
