# Supplementary material for: Contemporary Evolution of an At‐Risk Stickleback Population During a Severe Drought
Source: Evol Appl. 2026 Jan 6;19(1):e70189. doi: 10.1111/eva.70189 (PMC12772980; doi:10.1111/eva.70189)
Supplement: Supplementary file 1 — Table S1: Percentage of individuals per sample with most common anterior lateral plate position (left side) phenotypes (see Figure 2; panel A for depiction of plate position). Plate no. refers to the total number of lateral plates present (left side only) and plate position refers to the position where the plates are present (e.g., “5,6,7” means that fish have lateral plates present in positions 5, 6, and 7). Pre‐drought samples combine fish collected in years 2007, 2012, and 2013. Table S2: Planned contrasts (pre‐drought vs. post‐drought 2018; post‐drought 2018 vs. post‐drought 2022; post‐drought 2022 vs. outlet) from GLMs where trait is the repones variable, and sex and sample are the fixed effects. Figure S1: Timeline depicting sample sizes (above) per year (below) and samples used for whole‐genome pool‐sequencing. Figure S2: Allometrically size adjusted and z‐transformed trait measures per sample for females only. Error bar are 95% bootstrapped confidence intervals for the sample means. Statistical significance between samples is indicated by brackets: *** p < 0.001, ** p < 0.01, *p < 0.05. These p‐values are extracted from GLMs with sex and sample as predictor variables. Only comparisons of interest are depicted: Pre‐drought versus Post‐drought 2018, Post‐drought 2018 versus Post‐drought 2022 and Post‐drought 2022 versus Outlet. Dorsal spine 1 was absent in some populations (not included in figure): Pre‐drought: n = 4; Post‐drought 2018: n = 4, Post‐drought 2022: n = 4; Outlet: n = 3. Figure S3: Allometrically size adjusted and z‐transformed trait measures per sample for males only. Error bar are 95% bootstrapped confidence intervals for the sample means. Statistical significance between samples is indicated by brackets: *** p < 0.001, ** p < 0.01, *p < 0.05. These p‐values are extracted from GLMs with sex and sample as predictor variables. Only comparisons of interest are depicted: Pre‐drought versus Post‐drought 2018, Post‐drought 2018 versus Post‐drought 202 [file EVA-19-e70189-s001.docx]

SUPPLEMENTAL MATERIAL

**Table S1.** Percentage of individuals per sample with most common anterior lateral plate position (left side) phenotypes (see Fig. 2; panel A for depiction of plate position). Plate no. refers to the total number of lateral plates present (left side only) and plate position refers to the position where the plates are present (e.g. “5,6,7” means that fish have lateral plates present in positions 5, 6, and 7). Pre-drought samples combine fish collected in years 2007, 2012, and 2013.

|  |  | Lake (pre-drought) | |  | Lake (2018) | |  | Lake (2022) | |  | Outlet (2022) | |
| --- | --- | --- | --- | --- | --- | --- | --- | --- | --- | --- | --- | --- |
| Plate no. | Plate position | Females | Males |  | Females | Males |  | Females | Males |  | Females | Males |
| 0 | 0 | 38 | 13 |  | 6 | 7 |  | 7 | 9 |  | 55 | 20 |
| 1 | 6 | 9 | 13 |  | 10 | 6 |  | 2 | 2 |  | 5 | 10 |
|  | 7 | 2 | 0 |  | 3 | 2 |  | 0 | 5 |  |  |  |
| 2 | 5,6 | 9 | 0 |  | 10 | 6 |  | 4 | 4 |  | 5 | 0 |
|  | 6,7 | 4 | 2 |  | 7 | 9 |  | 14 | 16 |  | 5 | 0 |
| 3 | 5,6,7 | 7 | 0 |  | 13 | 10 |  | 10 | 15 |  |  |  |
|  | 6,7,8 |  |  |  | 0 | 1 |  | 1 | 1 |  |  |  |
| 4 | 4,5,6,7 |  |  |  | 1 | 0 |  | 0 | 1 |  |  |  |
|  | 5,6,7,8 |  |  |  | 3 | 2 |  | 0 | 3 |  |  |  |

**Table S2.** Planned contrasts (pre-drought vs post-drought 2018; post-drought 2018 vs post-drought 2022; post-drought 2022 vs outlet) from GLMs where trait is the repones variable, and sex and sample are the fixed effects.

| **Trait** | **Contrast** | **Effect size** | **Lower CI** | **Upper CI** | **EMM** | **SE** | ***t* ratio** | ***p* value** | **R^2^  Sex** | **R^2^ Sample** |
| --- | --- | --- | --- | --- | --- | --- | --- | --- | --- | --- |
| *Body depth* | Pre vs 2018 | -0.042 | -0.405 | 0.322 | 0.039 | 0.172 | 0.227 | 0.821 | 0.091 | 0.001 |
|  | 2018 vs 2022 | -0.077 | -0.368 | 0.214 | 0.072 | 0.138 | 0.519 | 0.604 | 0.213 | 0.001 |
|  | 2022 vs Out | 0.137 | -0.353 | 0.626 | -0.128 | 0.232 | -0.550 | 0.583 | 0.172 | 0.002 |
| *AP length* | Pre vs 2018 | 0.388 | 0.025 | 0.752 | -0.380 | 0.180 | -2.103 | **0.036** | 0.003 | 0.031 |
|  | 2018 vs 2022 | -0.130 | -0.421 | 0.161 | 0.127 | 0.145 | 0.879 | 0.380 | 0.001 | 0.005 |
|  | 2022 vs Out | -0.767 | -1.259 | -0.274 | 0.750 | 0.243 | 3.088 | **0.002** | 0.003 | 0.078 |
| *AP width* | Pre vs 2018 | -0.279 | -0.643 | 0.086 | 0.270 | 0.179 | 1.510 | 0.132 | 0.029 | 0.016 |
|  | 2018 vs 2022 | -0.246 | -0.537 | 0.045 | 0.239 | 0.143 | 1.666 | 0.097 | 0.015 | 0.016 |
|  | 2022 vs Out | -0.361 | -0.851 | 0.130 | 0.350 | 0.241 | 1.452 | 0.148 | 0.003 | 0.013 |
| *Jaw length* | Pre vs 2018 | 0.708 | 0.344 | 1.073 | -0.556 | 0.145 | -3.839 | **<0.001** | 0.338 | 0.094 |
|  | 2018 vs 2022 | -0.157 | -0.448 | 0.135 | 0.123 | 0.116 | 1.060 | 0.290 | 0.377 | 0.007 |
|  | 2022 vs Out | -0.709 | -1.201 | -0.217 | 0.556 | 0.195 | 2.854 | **0.005** | 0.419 | 0.072 |
| *PP length* | Pre vs 2018 | -0.182 | -0.545 | 0.182 | 0.177 | 0.180 | 0.984 | 0.326 | 0.004 | 0.012 |
|  | 2018 vs 2022 | 0.420 | 0.128 | 0.711 | -0.409 | 0.144 | -2.838 | **0.005** | 0.011 | 0.047 |
|  | 2022 vs Out | -0.941 | -1.432 | -0.451 | 0.917 | 0.242 | 3.790 | **<0.001** | 0.020 | 0.107 |
| *PP width* | Pre vs 2018 | 0.943 | 0.575 | 1.312 | -0.861 | 0.168 | -5.112 | **<0.001** | 0.003 | 0.160 |
|  | 2018 vs 2022 | 0.243 | -0.050 | 0.536 | -0.222 | 0.135 | -1.639 | 0.102 | <0.001 | 0.015 |
|  | 2022 vs Out | -1.007 | -1.497 | -0.516 | 0.919 | 0.227 | 4.048 | **<0.001** | <0.001 | 0.128 |
| *Dorsal 1* | Pre vs 2018 | -0.446 | -0.811 | -0.081 | 0.512 | 0.199 | 2.575 | **0.011** | <0.001 | 0.059 |
|  | 2018 vs 2022 | -0.132 | -0.423 | 0.160 | 0.139 | 0.153 | 0.905 | 0.366 | 0.001 | 0.005 |
|  | 2022 vs Out | 0.034 | -0.455 | 0.523 | -0.025 | 0.270 | -0.093 | 0.926 | <0.001 | <0.001 |
| *Dorsal 2* | Pre vs 2018 | -0.273 | -0.637 | 0.092 | 0.282 | 0.177 | 1.595 | 0.112 | 0.056 | 0.023 |
|  | 2018 vs 2022 | -0.695 | -0.987 | -0.404 | 0.652 | 0.139 | 4.700 | **<0.001** | 0.013 | 0.113 |
|  | 2022 vs Out | 0.254 | -0.236 | 0.743 | -0.238 | 0.232 | -1.026 | 0.306 | 0.013 | 0.006 |
| *Pelvic spine* | Pre vs 2018 | -0.168 | -0.532 | 0.196 | 0.168 | 0.182 | 0.924 | 0.356 | 0.055 | 0.009 |
|  | 2018 vs 2022 | -0.412 | -0.704 | -0.121 | 0.399 | 0.144 | 2.776 | **0.006** | 0.018 | 0.039 |
|  | 2022 vs Out | 0.715 | 0.225 | 1.204 | -0.692 | 0.241 | -2.864 | **0.005** | 0.005 | 0.054 |
|  |  |  |  |  |  |  | *z* ratio |  |  |  |
| *Lateral plates* | Pre vs 2018 | -0.908 | -1.275 | -0.541 | 0.908 | 0.186 | 4.871 | **<0.001** | <0.001 | 0.199 |
|  | 2018 vs 2022 | -0.002 | -0.211 | 0.206 | 0.002 | 0.106 | 0.020 | 0.984 | 0.001 | <0.001 |
|  | 2022 vs Out | 1.709 | 0.951 | 2.466 | -1.709 | 0.386 | -4.427 | **<0.001** | 0.002 | 0.237 |

Figure S1. Timeline depicting sample sizes (above) per year (below) and samples used for whole-genome pool-sequencing.


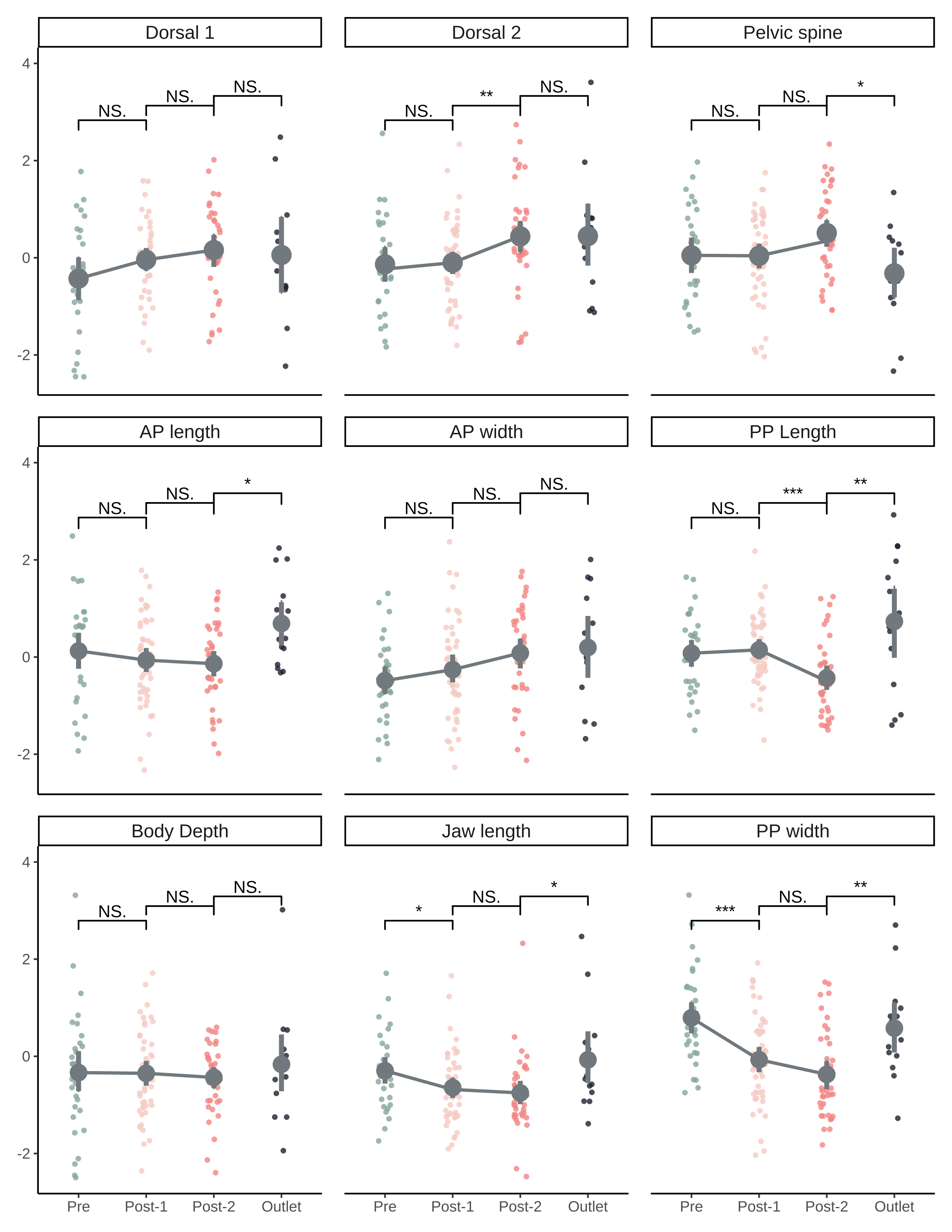


**Figure S2.** Allometrically size adjusted and z-transformed trait measures per sample for females only. Error bar are 95% bootstrapped confidence intervals for the sample means. Statistical significance between samples is indicated by brackets: *** p<0.001, ** p<0.01, *p<0.05. These p-values are extracted from GLMs with sex and sample as predictor variables. Only comparisons of interest are depicted: Pre-drought vs. Post-drought 2018, Post-drought 2018 vs. Post-drought 2022, and Post-drought 2022 vs. Outlet. Dorsal spine 1 was absent in some populations (not included in figure): Pre-drought: n=4; Post-drought 2018: n= 4, Post-drought 2022: n=4; Outlet: n=3.


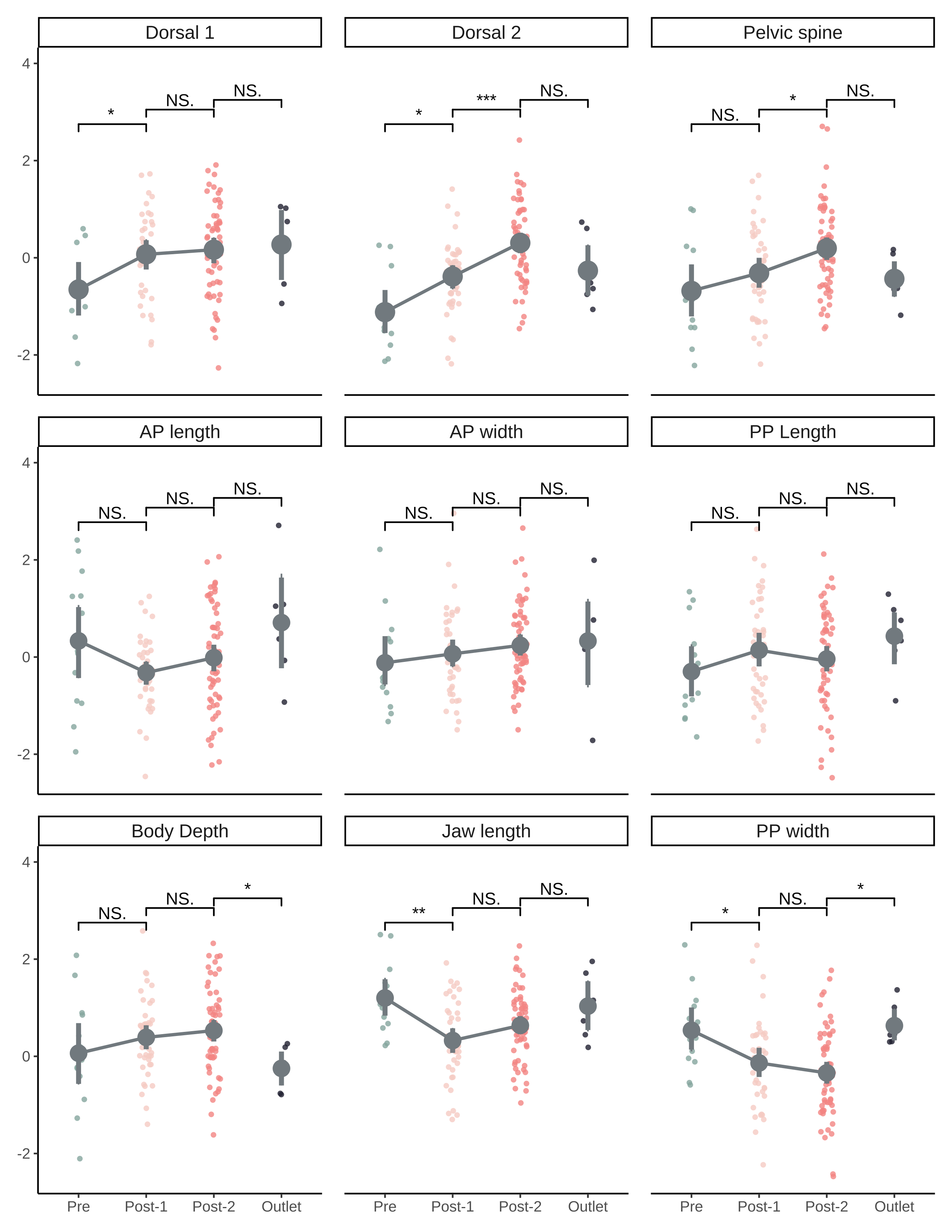


**Figure S3.** Allometrically size adjusted and z-transformed trait measures per sample for males only. Error bar are 95% bootstrapped confidence intervals for the sample means. Statistical significance between samples is indicated by brackets: *** p<0.001, ** p<0.01, *p<0.05. These p-values are extracted from GLMs with sex and sample as predictor variables. Only comparisons of interest are depicted: Pre-drought vs. Post-drought 2018, Post-drought 2018 vs. Post-drought 2022, and Post-drought 2022 vs. Outlet. Dorsal spine 1 was absent in some populations (not included in figure): Pre-drought: n=1; Post-drought 2018: n= 2, Post-drought 2022: n=4; Outlet: n=0.
